# Effects of MBSR therapy on negative emotions, fatigue, and sleep quality in “post-ICU patients”: A randomized controlled clinical trial protocol

**DOI:** 10.1097/MD.0000000000028331

**Published:** 2022-01-07

**Authors:** Yu Chen, Renying Wang, Jie Yu, Liping Zhu, Yiming Lu, Xueqin Deng

**Affiliations:** Department of Emergency, Ruijin Hospital, Shanghai Jiao Tong University School of Medicine, Shanghai, China.

**Keywords:** general ward, ICU survivor, mindfulness-based stress reduction, protocol, randomized controlled trial

## Abstract

**Background:**

Survivors of intensive care unit (ICU) transfer to the common ward are often accompanied by psychological distress, negative emotions, fatigue, and sleep disturbances that affect recovery. Mindfulness-based stress reduction (MBSR) has achieved reliable results in improving physical and mental health. However, no clinical study has been conducted to evaluate the effects of MBSR on negative emotions, fatigue and sleep quality of patients who survived ICU and were transferred to general wards.

**Methods:**

This is a prospective randomized controlled trial (RCT) examining the effects of MBSR on negative emotions, fatigue, and sleep quality in inpatients transferred from ICU to general ward. Participants were randomly divided into the treatment group and the control group in a ratio of 1:1. On the basis of the same nursing plan and health education, the treatment group received MBSR therapy, while the control group received no other interventions, and all the patients were followed up for 3 months after 2 weeks of continuous treatment. The indicators included negative mood indicators [Self-rating Depression Scale (SDS) and Self-Rating Anxiety Scale (SAS)], fatigue index [Fatigue Severity Scale (FSS) and Brief Fatigue Inventory (BFI)], and sleep quality index [Pittsburgh Sleep Quality Index (PSQI)]. Finally, SPSS 20.0 software was used for statistical analysis of the data.

**Discussion:**

This study will evaluate the effects of MBSR on negative emotions, fatigue, and sleep quality in hospitalized patients transferred from ICU to general ward. The results of this study will provide a reference for MBSR to improve psychological distress in ICU survivors transferred to general ward.

**Trial registration:**

This study protocol was registered in the Open Science Framework (OSF) (registration number: DOI 10.17605/OSF.IO/PD7SU).

## 1 Introduction

Most patients admitted to the intensive care unit (ICU) are life-threatening and accompanied by multiple organ failure, requiring specialized nurses with strong professional ability to care for them. After a series of treatment and fine nursing, they will be transferred to the general ward for follow-up diagnosis and treatment after their condition is relieved. Patients’ transferring from ICU to general ward is a transition from advanced care state to low-level care state, which includes the loss of one-to-one care and the reduction of monitoring instruments and close attention,^[1]^ so patients may have corresponding adverse consequences. Some scholars have called the impact of such changes in environment and care relationship on patients and their families as “transition barriers,”^[2]^ and ICU survivors become vulnerable groups after being transferred to the common ward.^[3]^ Of the more than 2 million ICU survivors each year, up to 75% will experience months or even years of psychological distress.^[4]^ A number of studies have indicated that ICU survivors will develop multiple negative emotions, including anxiety, depression, post-traumatic syndrome, and other emotions, and such negative symptoms will last for more than 1 year.^[5–7]^ At present, clinical worker pay more attention to the safety of transition from ICU to general ward,^[8,9]^ the professionalism and continuity of nursing,^[10–12]^ and the care of family members,^[13]^ while ignoring the patients’ own physical and mental state regulation. In clinical practice, many ICU survivors will show negative emotions, fatigue and sleep disorders after being transferred to the common ward,^[14]^ and these symptoms cannot be completely solved by improving the external environment. Therefore, an appropriate psychological decompression program is very important for patients.

Mindfulness is a learned practice of non-judgmental awareness that aims to alleviate distress by uncoupling emotional reactions and habitual behavior from unpleasant symptoms, memories, thoughts, and emotions.^[15,16]^ Meta-analyses show that mindfulness-based stress reduction (MBSR) can effectively relieve emotional symptoms and improve overall function.^[17]^ Currently, MBSR has been widely used in post-traumatic stress disorder,^[18]^ cancer,^[19,20]^ and other diseases, and the results show that MBSR has a positive effect on improving negative emotions and sleep disorders.^[21,22]^ However, for ICU survivors, this therapy is only applied to discharged patients at present.^[4,7]^ Although the feasibility and acceptability of this therapy for ICU survivors and positive effects on patients’ psychological distress and physical symptoms have been confirmed, the family environment is completely different from the general ward environment. There is currently a lack of standard clinical studies to evaluate the effect of MBSR on inpatients transferred from ICU to general wards. We will investigate the effects of MBSR on negative emotions, fatigue, and sleep quality in post-ICU patients in a prospective randomized controlled study.

## 2 Materials and methods

### 2.1 Study design

**Figure 1:**
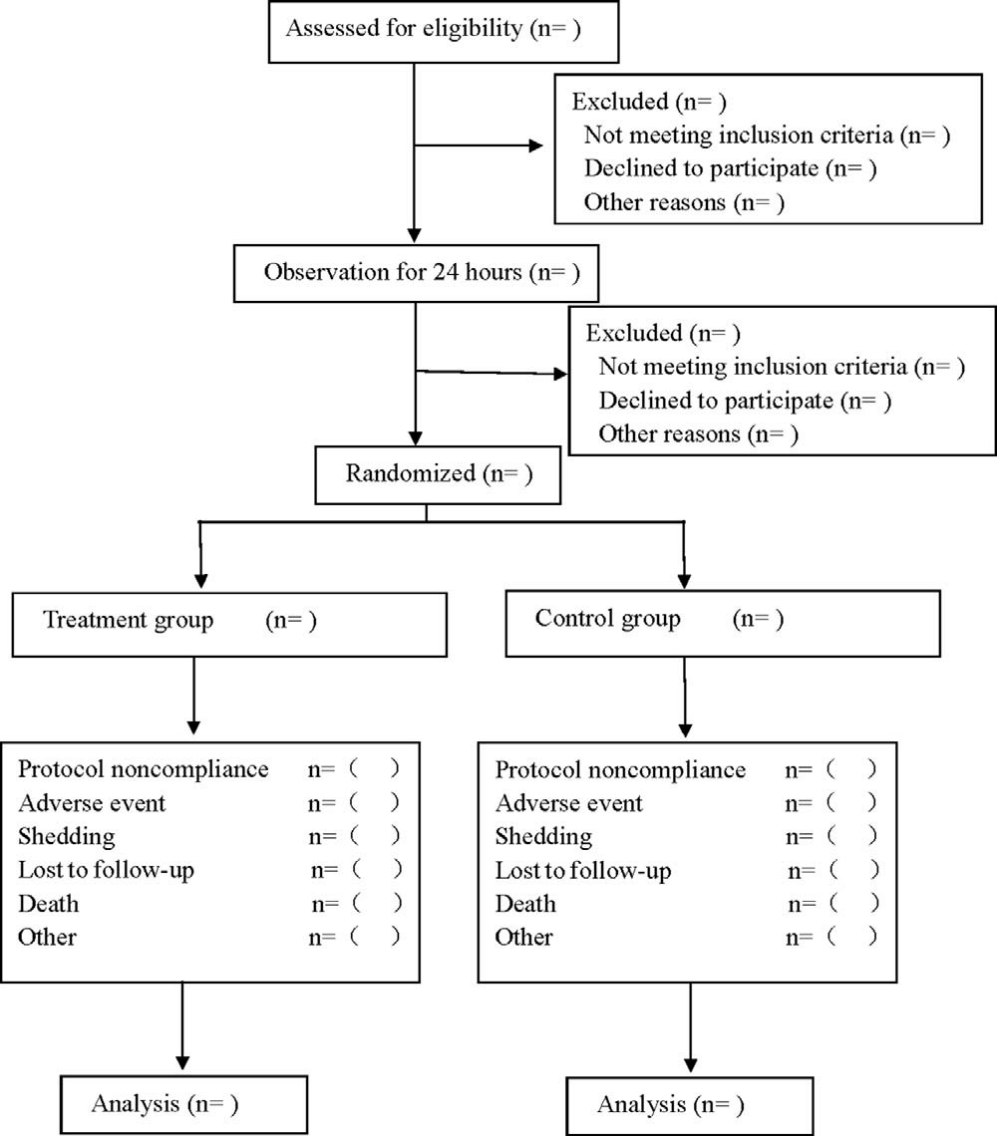
Flow diagram.

This is a prospective randomized controlled study examining the effects of MBSR on negative emotions, fatigue, and sleep quality in inpatients transferred from ICU to general ward. Eligible patients had a 24-hour observation period after being transferred from ICU to the general ward. Patients who still met the study criteria after the observation period were randomly divided into treatment group and control group in a 1:1 ratio. The treatment group received MBSR therapy, while the control group received routine health education. Follow-up was conducted for 3 months after 2 weeks of continuous treatment. The flow diagram is shown in Figure 1, and the study schedule is summarized in Table 1.

**Table 1:** Study schedule.

Stage project	Screening period	Treatment Period		Follow-up		
	Baseline	1-wk	2-wk	1-mo	2-mo	3-mo
Record fill	√					
Fulfill inclusion criteria and exclusion criteria	√					
Sign informed consent	√					
Random allocation	√					
Treatment	√	√	√			
Effectiveness observation						
SDS	√		√	√		√
SAS	√		√	√		√
FSS	√		√	√		√
BFI	√		√	√		√
PSQI	√		√	√		√
Safety evaluation						
Record of adverse event		√	√	√	√	√

### 2.2 Ethics and registration

This research protocol were conducted in accordance with the Declaration of Helsinki and Ethical Guidelines for Clinical Research. The protocol was also in strict accordance with the latest Consolidated Standards of Reporting Trials (CONSORT 2017) and Standard Protocol Items: Recommendations for Interventional Trials (SPIRIT) 2013 Statement. This study has been approved by our Clinical Research Ethics Committee and registered with the Open Science Framework (OSF) (registration number: DOI 10.17605/OSF.IO/PD7SU). All patients and their families signed a written informed consent form before the start of the study, and they could discontinue or withdraw from the study at any time during the study.

### 2.3 Patients

#### 2.3.1 Inclusion criteria

(1) Age ≥18 years;
(2) Stay in ICU for more than 12 hours and for the first time;
(3) The primary disease was improved and the patient's condition was stable;
(4) The patient's cognitive status was intact, and there was no history of cognitive impairment, such as dementia, and no obvious cognitive impairment at present (impairment defined as ≥3 errors on the Callahan cognitive status screen);
(5) No serious or persistent mental illness (such as bipolar disorder, schizophrenia, etc)
(6) Subjects agreed to participate in this study and signed informed consent.

#### 2.3.2 Exclusion criteria

(1) The patient would undergo complex medical care again in the near future (e.g., planned surgery, disruptive chemotherapy/radiation regiments, etc);
(2) Patients unable to complete the study procedures in the study plan;
(3) Patients who have participated in or were currently participating in other clinical trials within the past 1 month.

#### 2.3.3 Shedding standard

(1) Adverse events (AEs) or severe adverse events (SAEs) occurred, and researchers should suspend or stop the study and give corresponding treatment according to their disease judgment;
(2) The patient's condition deteriorated during the study period, and although the prescribed treatment or follow-up had not been completed, the subject was asked to withdraw from the study and received other effective treatment in order to protect the subject;
(3) A subject who, for whatever reason, was unwilling or unable to continue the clinical trial and requested the investigator to withdraw from the trial.

For patients who dropped out of the trial or were lost to follow-up, researchers should actively take measures to complete the last test as far as possible, so as to analyze the efficacy and safety of the test and take corresponding treatment measures. All cases of detachment should be recorded on the Case Report Form (CRF), fill in the cause of case shedding.

### 2.4 Sample size

Depression is the most common negative emotion in ICU survivors. The sample size estimation of this study was based on the mean and standard deviation of self-rating Depression Scale (SDS) scores of patients 2 weeks after treatment. Referring to the results of the preliminary experiment, it was 48.76 ± 5.29 in the treatment group and 51.66 ± 4.76 in the control group, set α = 0.025, unilateral test, β = 0.80. The PASS15.0 software calculated that 49 participants were required for each group, the estimated drop-out rate was 10%, and 55 patients would be enrolled in each group.

### 2.5 Randomization and blinding

Patients who meet the criteria were randomly assigned to either the treatment group or the control group in a 1:1 ratio. Randomization procedures were performed using SAS 9.3 software (SAS Institute, Cary, NC) by independent statisticians not involved in trial implementation or statistical analysis using a centralized network-based randomization tool. The research assistant entered patient information on a tablet computer and was assigned a random number to complete the random assignment. Due to the particularity of the intervention, the distribution results were known to the patients and the principal investigator, but not to the assistant and statistician responsible for data collection and analysis throughout the study.

### 2.6 Interventions

Both groups received the same nursing program and health education, including disease knowledge introduction, medication guidance, diet guidance, rehabilitation training and so on. The control group had no other special intervention measures. The treatment group received MBSR training on the basis of nursing program and health education as follows: MBSR training group was established, consisting of 2 responsible nurses, 4 specialist nurses, 1 psychologist, and 2 attending physicians. The responsible nurse and specialist nurse were responsible for the specific implementation of the program, and the psychologist and physician were responsible for the coping strategies of patients with adverse reactions during the implementation of the program. Team members must receive special training organized by the head nurse and pass the examination; Health education: The responsible nurse introduced the MBSR training mode in detail, including the theoretical knowledge, efficacy and precautions of MBSR training, to ensure that each patient is clear and master the training procedure; The responsible nurse guided and supervised the patients with mindfulness training, including body scan training, mindfulness breathing training, mind change awareness training, mindfulness eating awareness training, mindfulness meditation training (see Table 2).

**Table 2:** Contents of MBSR training.

Items	Contents
Body scan training	The patient was placed in a sitting or supine position, with the eyes naturally closed and the whole body relaxed. Guide the patient to perceive changes from the feet to other parts of the body.
Mindfulness breathing training	In a quiet and comfortable environment, the patient was aware of changes in respiratory airflow. In case of adverse emotions or other distractions, the patient was guided to concentrate and adjust breathing, and the patient was informed that adverse emotions could be removed. When the patient's attention was refocused on changes in respiratory airflow, the patient was instructed to feel changes in abdominal muscles.
Mind change awareness training	When patients appeared nervous, anxiety, depression, anxiety and other emotions, ask patients to recall the mood of pleasure and happy time, and feel the good feeling at that time, and then return to that kind of uneasy mood, to detect how the uneasy mood is generated, and how to overcome and face.
Mindfulness eating awareness training	Before eating, the patient was guided to put hands together and feel the beauty of food. When eating, refrain from speaking and carefully experience the pleasant mood of food being digested and absorbed in the gastrointestinal tract. At the same time, ask patients to feel the quiet and harmonious environment around them.
Mindfulness meditation training	Patients closed their eyes, relaxed, and recited positive words such as “I am the best” and “I am very happy.”

### 2.7 Outcomes

(1) Negative emotion evaluation: Self-rating Depression Scale (SDS) and Self-rating Anxiety Scale (SAS) were used to measure patients’ negative emotions.^[23]^ SDS and SAS were used to measure patients’ depression and anxiety respectively, both of which contain 20 items, with higher scores indicating more severe symptoms.
(2) Evaluation of fatigue: the Fatigue Severity Scale (FSS)^[24]^ and Brief Fatigue Inventory (BFI)^[25]^ were used to evaluate the fatigue degree of patients. Both FSS and BFI consist of 9 items, with higher scores indicating heavier fatigue.
(3) Evaluation of sleep quality: Pittsburgh Sleep Quality Index (PSQI) was used to evaluate the sleep condition of patients, including sleep quality, time to fall asleep, sleep duration, sleep efficiency, hypnotic drugs, sleep disorders, and daytime function. The total score is 21, and the higher the score is, the worse the sleep quality is.

Two research assistants will collect study data based on the above outcome measures at baseline and 2 weeks later, and at the end of the first and third month of follow-up, the research assistants will collect study data from patients in the outpatient clinic or by telephone (patients cannot attend the outpatient clinic due to objective reasons). Two research assistants would not know the results of randomization.

### 2.8 Data management and quality control

Any modifications or changes to the protocol would be re-approved through the formal procedures of our Ethics Committee. The safety Monitoring Committee would periodically review the progress of the research and ensure that standard procedures are followed. Study data were collected and recorded in CRF by trained investigators. In order to ensure the reliability of data, all of the patient's personal information were collected and stored in a separate storage. Access to the storage room was limited to the researchers of the research team, and data access must be carried out by 2 or more members to protect the confidentiality of the test before, during, and after the test. All patient information were disclosed and transmitted without the patient's written permission.

### 2.9 Statistical analysis

Efficacy evaluation was determined by the Full Analysis Set (FAS) and Per-Protocol Set (PPS), and the safety assessment were based on the Safety Set (SS). Statistical evaluation of FAS would follow intent-to-treat (ITT) principles. The last observation carried forward (LOCF) method was used to estimate missing values of major variables. The collected data were analyzed by SPSS 20.0 software. Chi-square test was used for counting data. Mean ± standard deviation ($\bar{x}\pm S$) was used for measurement data, independent sample *t* test was used for normal distribution, and Mann–Whitney *U* test was used for skewness distribution. The difference was considered statistically significant when *P* < .05.

## 3 Discussion

With the continuous progress of diagnosis and treatment technology, the survival rate of ICU patients is gradually improving, and the probability of patients being transferred out of ICU is also increasing.^[4]^ Patients who are transferred from the ICU to the general ward receive either primary care or advanced care; however, there are differences between ICU and general ward in terms of equipment, specialized experience of medical staff and care ability of family members, at the same time, patients and their families need to readjust to the environment. On the basis of the above reasons, the continuous care of patients will be affected to some extent and the nursing risk will increase.^[3]^ These factors also increase the risk of severe and persistent psychological distress for ICU survivors.

Negative emotions, mainly anxiety and depression, as well as fatigue and sleep disturbances, are the main psychological distress faced by ICU survivors,^[26]^ and these disturbances usually interact with and coexist with each other,^[27]^ and adversely affect the recovery of patients.^[26]^ MBSR has achieved significant efficacy in the regulation of physical and mental health in a variety of diseases.^[18–20]^ MBSR is currently used primarily for discharged ICU survivors and is administered via multiple mobile programs or telephone,^[4,7,16]^ whereas standard MBSR, which typically provides face-to-face training in a group setting,^[28]^ is controversial for patients moving from ICU to general ward. Therefore, we intended to investigate the effects of MBSR on negative emotions, fatigue, and sleep quality in hospitalized patients transferred from ICU to general ward in a prospective randomized controlled trial.

There are also some shortcomings in this study. First, MBSR in this study was only implemented during hospitalization in the general ward, so the intervention time was only 2 weeks. Second, this study is a single-center study, and the patients included in the study may be regionalized. Finally, as the family atmosphere of different patients after discharge may have an impact on patients’ psychology, it may affect the follow-up results of the study.

## Author contributions

**Conceptualization:** Yu Chen and Renying Wang

**Data curation:** Yu Chen and Jie Yu

**Formal analysis:** Liping Zhu and Yiming Lu

**Funding acquisition:** Xueqin Deng

**Software:** Liping Zhu and Yiming Lu

**Supervision:** Yu Chen and Jie Yu

**Writing – original draft:** Xueqin Deng and Renying Wang

**Writing – review & editing:** Xueqin Deng and Yu Chen

**Conceptualization:** Yu Chen.

**Data curation:** Jie Yu.

**Formal analysis:** Liping Zhu.

**Funding acquisition:** Xueqin Deng.

**Software:** Yiming Lu.

**Writing – original draft:** Renying Wang.
